# Patterns of Post-Glacial Genetic Differentiation in Marginal Populations of a Marine Microalga

**DOI:** 10.1371/journal.pone.0053602

**Published:** 2012-12-31

**Authors:** Pia Tahvanainen, Tilman J. Alpermann, Rosa Isabel Figueroa, Uwe John, Päivi Hakanen, Satoshi Nagai, Jaanika Blomster, Anke Kremp

**Affiliations:** 1 Marine Research Centre, Finnish Environment Institute, Helsinki, Finland; 2 Department of Environmental Sciences, University of Helsinki, Helsinki, Finland; 3 LOEWE Biodiversity and Climate Research Centre, Frankfurt, Germany; 4 Senckenberg Research Institute and Nature Museum, Frankfurt, Germany; 5 Aquatic Ecology, Lund University, Lund, Sweden; 6 Instituto Español de Oceanografía, Vigo, Spain; 7 Alfred Wegener Institute for Polar and Marine Research, Bremerhaven, Germany; 8 Research Centre for Aquatic Genomics, National Research Institute of Fisheries Science, Yokohama, Japan; Uppsala University, Sweden

## Abstract

This study investigates the genetic structure of an eukaryotic microorganism, the toxic dinoflagellate *Alexandrium ostenfeldii*, from the Baltic Sea, a geologically young and ecologically marginal brackish water estuary which is predicted to support evolution of distinct, genetically impoverished lineages of marine macroorganisms. Analyses of the internal transcribed spacer (ITS) sequences and Amplified Fragment Length Polymorphism (AFLP) of 84 *A. ostenfeldii* isolates from five different Baltic locations and multiple external sites revealed that Baltic *A. ostenfeldii* is phylogenetically differentiated from other lineages of the species and micro-geographically fragmented within the Baltic Sea. Significant genetic differentiation (*F*_ST_) between northern and southern locations was correlated to geographical distance. However, instead of discrete genetic units or continuous genetic differentiation, the analysis of population structure suggests a complex and partially hierarchic pattern of genetic differentiation. The observed pattern suggests that initial colonization was followed by local differentiation and varying degrees of dispersal, most likely depending on local habitat conditions and prevailing current systems separating the Baltic Sea populations. Local subpopulations generally exhibited low levels of overall gene diversity. Association analysis suggests predominately asexual reproduction most likely accompanied by frequency shifts of clonal lineages during planktonic growth. Our results indicate that the general pattern of genetic differentiation and reduced genetic diversity of Baltic populations found in large organisms also applies to microscopic eukaryotic organisms.

## Introduction

Due to their small body size, large population sizes, high rates of predominantly asexual reproduction and their open and seemingly homogenous pelagic habitat, aquatic microorganisms have been critically discussed to possess more or less cosmopolitan distributions and do not show biogeographies as found in macroorganisms [1]. Studies, ranging from bacteria to small metazoans, however, challenge Beijerinck’s “everything is everywhere” hypothesis and its contemporary variants [2]. Molecular data indicate that the distribution patterns of aquatic microorganisms reflect historical, ecological and local conditions [2] and that most species consequently should be considered systems of genetically structured components, metapopulations, rather than single panmictic populations [3].

Recent work on the genetic structure of eukaryotic microalgae revealed that most of the investigated species are highly structured at and below the population level [4], [5], [6]. Such evidence of genetic diversity and differentiation indicates that the general mechanisms affecting gene flow and facilitating adaptive divergence are also effective in these organisms. Microalgal diversity patterns have been related to geographic distance and isolation [7], landscape or seascape topography and current systems [8], environmental gradients [9], [10], as well as reproduction and life cycle strategies [11], [12], [6].

As a historically young aquatic system with steep environmental gradients, the Baltic Sea is a particularly intriguing model system for the study of genetic differentiation and species population structure. Marine organisms most likely colonized this system after the postglacial opening to the North Sea approximately 8000–3000 BP [13]. Much of the postglacial diversity has disappeared since then, as a result of the declining salinities: only a few marine species were able to adapt to the permanently low salinities of <10 psu (Practical Salinity Scale). Together with more recently introduced fresh- and brackish water species they now constitute a significantly impoverished Baltic flora and fauna.

The populations living in the Baltic Sea are considered peripheral to their marine temperate sister populations. They inhabit a geographically and ecologically marginal environment where isolation and exposure to harsh physical conditions, environmental gradients, and anthropogenic impact represent strong selection regimes [14]. Due to bottleneck effects and specific life history characteristics, such peripheral populations are generally predicted to have reduced genetic diversity [15], [16]. Recent meta-analyses of population genetic data confirmed that Baltic populations of marine macroorganisms are generally less diverse than oceanic populations [14]. These analyses also emphasized that Baltic populations of most investigated species were genetically strongly differentiated from North Sea and Atlantic populations. The observed patterns of differentiation and reduced genetic diversity are considered to be caused by geographic isolation, environmental selection pressures [17], and vestigilization of sexuality and clonality [18], [19].

Given the importance of genetic diversity for ecosystem function and stability as well as for adaptation to environmental change [20], [21], genetic diversity patterns have been particularly intensively investigated in ecologically important key players of the Baltic ecosystem, such as fish species, benthic bivalves and macrophytes. It has been suggested that the high degree of genetic endemism and the reduced genetic variation found in these key organisms e.g. [22], [23] may negatively affect their response to environmental change [19]. In contrast to macroscopic organisms, very little is known to date about the population structure of Baltic planktonic microorganisms. Whereas some studies cover aspects of genetic diversity patterns in prokaryotic microbes [24], [25] to our knowledge not a single study addresses these issues in eukaryotic microbial populations in the Baltic Sea, despite their important role as primary producers at the base of the aquatic food web. If the general pattern of distinct lineages and reduced genetic diversity seen in Baltic macroorganisms would occur also in key phytoplankton, this could have significant ecological implications.

To gain insights into genetic diversity patterns of Baltic phytoplankton populations and the relative importance of isolation, local adaptation and specific life history traits as underlying mechanisms, we investigated the genetic structure of the dinoflagellate *Alexandrium ostenfeldii* (Paulsen) Balech et Tangen in the Baltic Sea. This dinoflagellate is part of the *A. ostenfeldii/peruvianum* species complex with unresolved species boundaries, comprising different morphotypes. Typically a rare background species that co-occurs at relatively low numbers with other phytoplankton [26], [27], [28] the widely distributed *A. ostenfeldii* forms dense toxic blooms at the Baltic Sea coasts [29]. Like many other dinoflagellates, *A. ostenfeldii* produces dormant resting cysts as a part of its meroplanktonic life cycle [30]. The cysts represent a source of genetic recombination, as they are, at least in part, sexual products of gamete fusion, and planozygote encystment [31].

By analyzing internal transcribed spacer (ITS) and 5.8S rDNA sequences and amplified fragment length polymorphism (AFLP) data of 84 isolates from 5 geographically separated bloom locations in the Baltic Sea and comparing them with data obtained from global isolates, we here address the following overarching question: Do metapopulations of marine protist species exhibit similar diversity profiles as metazoans in the Baltic Sea and to what extent is this due to Baltic Sea particularities? Specifically we investigate 1) the degree of genetic divergence of Baltic *A. ostenfeldii* populations from neighboring North Atlantic and other global populations of this species and 2) the genetic structure and patterns of genetic diversity of this species among and within its populations in the Baltic Sea.

## Materials and Methods

### Study Area

**Figure 1:**
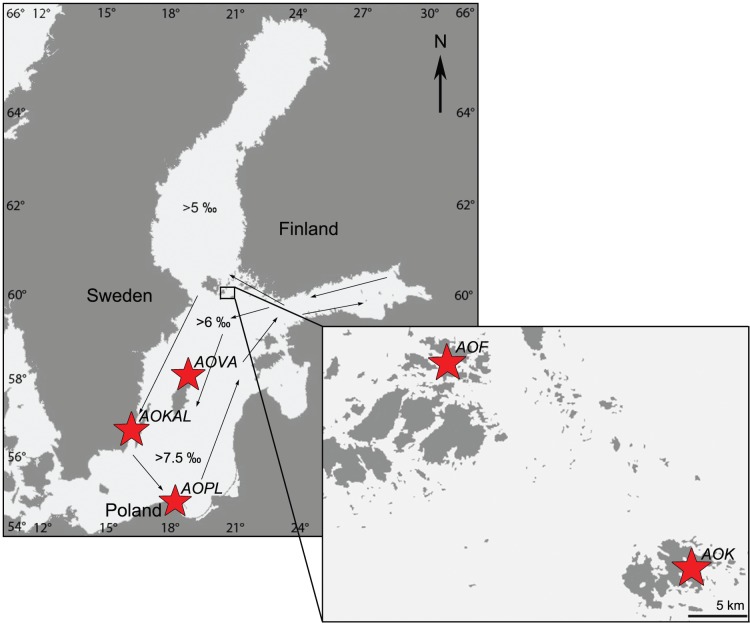
Map showing sampling sites around the Baltic Sea. Arrows indicate the directions of major currents according to [32]. AOF = Föglö; AOK = Kökar; AOVA = Valleviken, Gotland; AOKAL = Kalmar; AOPL = Puck Bay, Poland.

For this study we chose five coastal sites with known occurrences of *Alexandrium ostenfeldii* blooms (Fig. 1). The two northernmost sites were located in the Åland Sea, which is situated north of the Baltic Proper between the SW coast of Finland and the Stockholm archipelago (Sweden). The Åland archipelago consists of several larger and ca. 6500 small islands. The Föglö sampling station (N60°56′17, E20°32′44; at 1.5 m water depth) is located in a coastal embayment in the eastern archipelago area. The other Åland bloom site is located in a nearly enclosed shallow embayment in Kökar (N59°56′52, E20°58′58, 2 m water depth), an exposed island in the outer archipelago close to the border of Åland Sea and Baltic proper. Salinities at both Åland sites range between 6 and 6.5 psu. Sampling in the Åland area took place in March 2009 (Föglö) and 2010 (Kökar). The third bloom site, Valleviken, is a small, approximately 3 m deep sheltered harbor at the northern tip of Gotland (Sweden, N57°43′53, E18°57′23) in the central Baltic. Salinities here typically range between 6.5 and 7 psu. The southern bloom locations in the Kalmar sound (Sweden, N56°42′40, E16°21′45) and Puck Bay in the Gulf of Gdansk (Poland, N54°45′31, E18°30′32) both represent shallow (0.5–1.5 m depth), more open beaches with mostly coarse and sandy sediments. While salinities in Kalmar range between 7 and 7.5 psu, the Puck Bay site shares the salinity conditions with the Gotland site. Bloom sites in Gotland, Kalmar and Puck Bay were sampled in March 2009. Whereas the three central and southern Baltic bloom sites are connected by the central Baltic surface water circulation system, the Åland sites in the Northern Baltic are mainly influenced by eastern currents moving north- or westward and are thus separated from the other sites [32].

### Ethics Statement

No specific permits were required for the described field studies. The locations are not privately-owned or protected in any way. The field studies did not involve endangered or protected species.

### Culture Establishment and Survival

Sediment samples were collected from different locations using a gravity corer (LIMNOS, Turku, Finland), and subsamples of the uppermost (0–3 cm) layer were processed for cyst isolations. Single resting cysts were randomly picked from sonicated, sieved sediment slurries using a micropipette. Cysts were placed in individual wells of 24 well tissue culture plates, each filled with 2 mL of f/8–Si enriched natural sea water of a salinity of 6.5, and incubated at 16°C, 12∶12 light:dark cycle and 100 µmol photons m^–2 ^s^–1^. To ensure clonality (cysts may represent diploid hypnozygotes) a re-isolation step was performed: once germinated cells began to proliferate, single cells possessing one longitudinal flagellum (a characteristic feature of asexually growing, haploid dinoflagellate cells) were randomly picked and placed in a new culture well containing f/8-Si medium. Well established clonal cultures were transferred to vented 50 mL polycarbonate tissue culture flasks and maintained in f/2–Si culture medium at 16°C, 12∶12 light:dark cycle, and 100 µmol photons m^–2 ^s^–1^.

Isolation success ranged from 35 to 71% in the different populations (mean survival rate was 51.6%) and was generally higher in material from northern Baltic bloom sites than in central and southern Baltic material (Table S1). Isolation success largely depended on germination of the isolated cysts. Once re-isolated, most cultures started to proliferate rapidly. About 15 clonal strains of the established cultures were arbitrarily chosen from each population for genetic analyses, except for the Kalmar population, where cysts were scarce in the sediment samples and only 14 strains could be successfully established of which 12 were further used for genotyping (Table S1). A total of 84 strains were used for genotyping.

### DNA Extraction and Sequencing

Fifteen mL of clonal cultures were harvested from exponentially growing cultures. Strains were washed several times on 10 µm filters (Poretics polycarbonate membranes, Whatman) with sterile, filtered seawater (salinity 6) to remove possible bacteria. The cells were then rinsed into 15 mL centrifuge tubes containing 20 ml of sterile seawater and concentrated by centrifugation at 4000 g (15–30 min). The sample was re-centrifuged for 15 min at 14 000 g and the overlying liquid was aspirated to obtain a cell pellet. The cell pellets were disrupted by applying a pestle (Pellet Pestle Cordless Motor, Kontes Glass Company, Kimble), and DNA was extracted using a Plant Mini Kit (Qiagen) and purified with a PCR Template Purification Kit (Roche) according to the manufacturer’s instructions. DNA purity and concentration were measured with a NanoDrop ND-1000 (Thermo Scientific) and purified DNA samples were stored at –20°C until further processing.

All isolates were sequenced for the internal transcribed spacers (ITS-1, ITS-2 and 5.8 S rDNA gene, which were amplified using the primers and PCR settings specified by [33]. PCR reactions were performed in 25 µl reaction volume in PCR beads (IllustraPuReTaq Ready-to-go-PCR-beads, GE Healthcare) consisting 22 µl of sterile MQ water, 1 µl of each primer (10 µM) and 1 to 2 µl of DNA (about 50 ng). PCR products were purified using the GFX-PCR Purification Kit (Qiagen) following the manufacturer’s protocol. Sequencing reactions were carried out with the same forward or reverse primers [33] according to the protocol of Applied Biosystems with BigDye® Terminator v3.1 Cycle Sequencing Kit, and later purified in a Biomek® NXP Laboratory Automation Workstation (Beckman Coulter) according to the Agencourt® CleanSEQ kit protocol, and sequenced in an Applied Biosystems ABI3130XL Genetic Analyzer (16-capillaries) or ABI3730 DNA Analyzer (48-capillaries) (Applied Biosystems). The generated sequences are deposited in Genbank (for Accession numbers see Table S2).

### Phylogenetic Analysis

Prior to the phylogenetic analysis the 84 ITS-sequences obtained for each algal isolate (Table S2) were assembled and when necessary manually edited in Chromas Pro (Version 1.5.). Multiple sequence alignment was carried out in MAFFT (Multiple Alignment with Fast Fourier Transform) [34] in SeaView [35] using default settings. The data set for the alignment contained 18 ingroup sequences, including representatives of all divergent ITS lineages found in the 84 Baltic Sea isolates (Table S2), and had a total length of 573 bp. *Alexandrium minutum, A. tamutum* and *A. insuetum* were used as outgroups. The resulting alignment is deposited in a public web server (“PopSet” at ENTREZ), and will be also provided upon request.

To determine phylogenetic relationships of Baltic isolates within the *Alexandrium ostenfeldii/peruvianum* group we used a reduced set of the generated alignment containing all represented 24 haplotypes/ribotypes within the dataset. For a phylogenetic analyses based on Bayesian inference (BI), the software MrBayes v3.2 [36] was used with substitution model GTR+G [37], selected under the Bayesian Information Criterion (BIC) with jModelTest 0.1.1. [38]. As no specific knowledge on parameter priors was available, the default settings for prior distributions were used in all analyses. Two runs with four chains (one cold and three incrementally heated chains) were run for 15×10^6^ generations, sampling every 500 trees. In each run, the first 25% of samples were discarded as the burn-in phase. The stability of model parameters and the convergence of the two runs were confirmed using Tracer v1.5 [39].

Additionally, a maximum likelihood (ML) phylogenetic tree based on the reduced alignment was calculated with PhyML [40] using a BIO-NJ (neighbor-joining) tree as a starting tree and the GTR evolutionary model, with a gamma distribution parameter estimated from the data. Tree topology was supported with bootstrap values calculated with 1000 replicates.

Network analyses were done for ITS sequences using median joining in Network 4.6. [41] and statistical parsimony methods in TCS 1.21 [42]. Uncorrected genetic distances were calculated from ITS sequences (572 bp) with PAUP* 4.0a122 [43].

### AFLP Reactions

AFLP fragments were generated using the modified conditions previously described in [44] and [45], with the exception that DNA was digested for 17 hours at 37°C and dilutions for pre- and selective-amplification were done in five fold. Suitability of the restriction enzymes MseI and EcoRI was tested on a subset of samples by confirming the digestion success by each enzyme in separate reactions on agarose gels as in [46]. Five different primer pair combinations (Table S3), each containing two to three selective nucleotides, were used for selective-PCR reactions. Selective PCR-reactions were performed in PCR-conditions described in [44]. The final PCR products were diluted by 30 fold and separated by capillary electrophoresis with GeneScan™ 500 ROX (Applied Biosystems) as internal size standard, using an ABI 3730XL (Applied Biosystems) in Biotechnology Institute (Helsinki, Finland).

### AFLP Data Screening

Results from the capillary electrophoresis of a total of 84 individuals remained un-normalized when detecting size and height of peaks in the software GENEMAPPER 4.0 (Applied Biosystems) employing GENEMAPPER settings from [47]. The AFLP peak-height data of fragments from 100 to 500 bp was subsequently normalized and scored for the presence and absence using AFLPSCORE version 1.4 [47]. In this study all fragments were scored as present markers and in case of missing fragments, the bands were scored as absent. The locus selection thresholds and absolute phenotype-calling thresholds were set manually for each primer combination (locus-selection threshold being 150 and phenotype selection threshold 500); thereby ensuring mismatch-error-rates ranging from 2.5 to 6.9, the mean being 4.3. The reproducibility of AFLP markers was tested using the mismatch error rate between replicates (replicates covered 45% of the samples) from the same and different DNA extractions calculated with AFLPSCORE. The AFLP reproducibility was considered sufficient when the average mismatch error rate was below 0.05 [47].

### Genetic Structure and Diversity Analyses on AFLP Data

Genetic structure of Baltic Sea *A. ostenfeldii* was analysed by Bayesian probabilistic population assignment of AFLP multilocus genotypes in STRUCTURE 2.3.3. [48], [49]. Here, multilocus genotypes were assigned repeatedly to one of a predefined number of hypothetical subpopulations in a Markov chain Monte Carlo (MCMC) simulation aiming at maximizing the degree of genetic differentiation among hypothetical subpopulations by changing the assignment of individual multilocus genotypes in consecutive iterations [48]. The model used here (“admixture model”) estimated the proportion of each multilocus genotype to be derived from one of the hypothetical populations of origin, thereby allowing to detect specific signatures of admixture from the multilocus genotypes of individual strains. The model settings were such that allele frequencies were allowed to correlate, since allele frequencies are usually considered to be similar in different populations with recently shared ancestry and thus multilocus genotypes could be assigned even to weakly differentiated hypothetical subgroups [49]. We ran two sets of five runs as preliminary analysis during which either 100,000 or 500,000 iterations were used for initial burn-in period of the MCMC scheme, followed by either 100,000 or 500,000 iterations, respectively, during which parameter estimates were sampled. 20 independent runs with both parameter settings were performed for the AFLP dataset and for each number of hypothetical subpopulations, (*K*), ranging from one to seven. Convergence of parameter estimates was observed during runs with both settings and assessment of differences in the variation of the Log likelihood showed that parameters could not be estimated with less variation by extending the burn-in or the MCMC chain from which estimates were sampled. Results of STRUCTURE-analyses were evaluated following the Δ*K*-method of [50] in the utility STRUCTURE harvester version 0.6.7. [51] and also by following the strategy suggested in the software’s manual [52].

The scored data set was subsequently divided into putative populations based on their geographical origin to be used in ARLEQUIN 3.5 [53] to calculate genetic differentiation between populations by pair-wise *F*_ST_ values (p-values were determined using 10,000 permutations). The fixation index, *F*_ST_, is a measure of population differentiation based on the partitioning of genetic polymorphism, as e.g. represented in biallelic AFLP data. Values of *F*_ST_ range from 0 to 1, where zero implies no genetic differentiation and the value of one indicates that populations are completely differentiated.

The p-values were corrected for multiple pair-wise tests with Bonferroni and Šidák procedures [54]. The statistical power for the estimation of significance of pair-wise *F*_ST_ given the set of AFLP markers used in this study was assessed in POWSIM version 4.1 [55] for the population pair, which received lowest statistical support in the analysis of pair-wise differentiation (Kalmar-Gotland: *F*_ST_ = 0.07, p<0.05 after correction). As POWSIM is restricted to 50 loci, we selected those 50 AFLP loci with highest differentiating capacity (i.e. highest values of Nei’s gene diversity as determined for the combined data from Kalmar and Poland). In POWSIM the standard settings of 1,000 dememorizations (burn-in iterations), 100 batches with 1,000 iterations per batch were used.

Nei’s unbiased estimate of gene diversity across all loci [56] and the percentage of polymorphic loci (PPL) were determined for each sampling location and the combined Baltic Sea isolates using the R script AFLPdat [57]. For biallelic loci such as AFLP the unbiased estimate of Nei’s gene diversity approaches a value of ∼0.5 if both alleles are equally frequent, whereas lower values indicate that one of the two alleles becomes more frequent with a minimum value of 0 as only one allele is observed.

### AFLP Analyses of Multilocus Linkage Disequilibrium (LD)

Association analyses were applied on the multilocus genotype data to obtain information on the relative importance of asexual reproduction. The ‘standardized index of association’ *I* ^s^_A_ [58] takes increasing values in populations that deviate from panmixis e.g., due to clonality or admixture. Values of *I* ^s^_A_ and their significance were estimated by Monte Carlo simulation (10,000 resamplings without replacement) to assess if multilocus linkage disequilibrium was present within the populations of *A. ostenfeldii*. The calculations were performed in the software LIAN version 3.5 [59] using its web interface (http://adenine.biz.fh-weihenstephan.de/cgi-bin/lian/lian.cgi.pl).

## Results

### Phylogenetic Analyses

**Figure 2:**
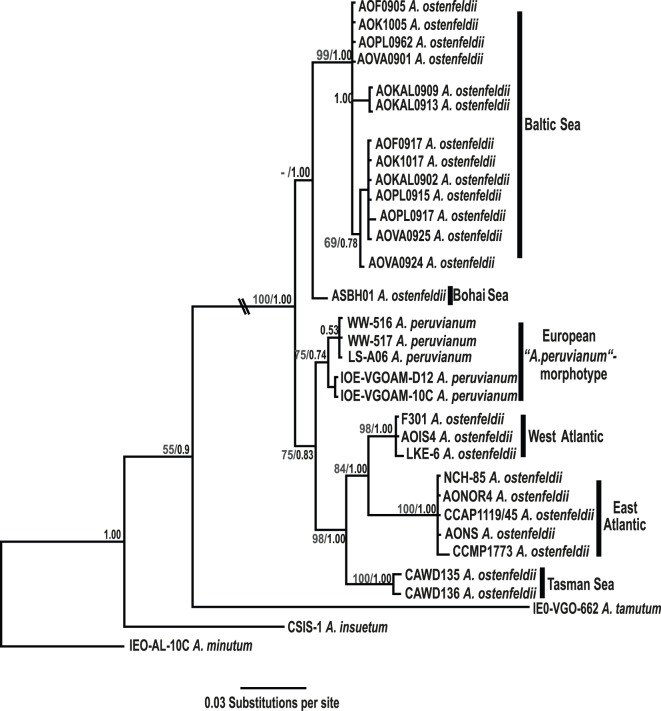
Phylogenetic tree for internal transcribed spacer (ITS-1 and ITS-2) and 5.8 rDNA sequences of *A. ostenfeldii* isolates from the Baltic Sea and other geographical locations as derived from Bayesian inference. Node labels correspond to posterior probabilities from Bayesian inference, BI, and bootstrap values from maximum likelihood, ML, analyses (ML/BI). For Baltic Sea strains isolated in this study codes begin with the location identifier: AOF = Föglö, Åland; AOK = Kökar, Åland; AOVA = Gotland; AOKAL = Kalmar; AOPL = Puck Bay, Poland.

The phylogenetic analyses (Fig. 2.) showed that Baltic strains of *Alexandrium ostenfeldii* form a monophyletic group. Within the Baltic clade the ITS sequence did not show high differentiation. Minor nucleotide changes were observed among some, but not all, isolates from Kalmar (difference being 0 to 3 nucleotides) (Table S4). While most of the isolates from the Baltic locations grouped into two close groups with high bootstrap and posterior probability values (ML 99%, BI 1.0). The Chinese strain from the Bohai Sea, ASBH01, seems most closely related to Baltic isolates, yet differing at 10 nucleotides. This strain forms the sister lineage to the monophyletic Baltic Sea clade in the phylogenetic tree derived from Bayesian inference, but was not recovered as such in the maximum likelihood based analysis (ML<50%, BI 1.0). All other *A. ostenfeldii/peruvianum* isolates fell into another, highly complex cluster that was further subdivided into several clades: one comprising strains belonging to the *A. peruvianum* morphotype (differing in the ITS region with about 14 to 16 nucleotides), and the other containing groups of strains from the West Atlantic, East Atlantic and the Tasman Sea.

**Figure 3:**
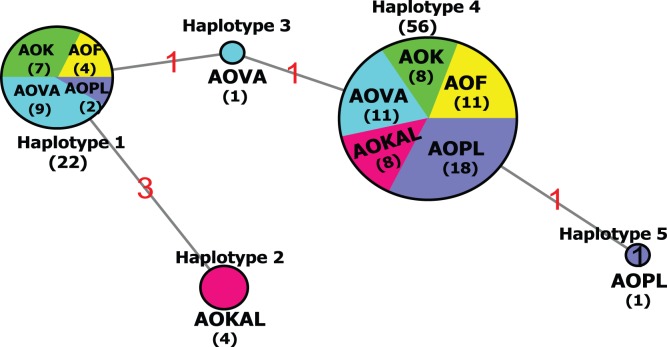
Haplotype network based on 84 ITS sequences: Mutation steps are shown in red. Number of analyzed strains is given inside the haplotypes.

Network analysis (Fig. 3) found five different ITS haplotypes with six mutation steps in the shortest tree. All populations shared the same main haplotype 4 (56 isolates), as well as all populations, except Kalmar, shared the second most common haplotype 1 (22 isolates), clearly showing that strong differentiation among Baltic Sea populations could not be observed from the ITS network. Only four isolates from Kalmar, one isolate from Puck Bay, Poland and one from Gotland shared unique haplotypes (haplotype 2, 3 and 5).

Uncorrected genetic distance values calculated from ITS sequences (Table S4) among divergent Baltic Sea strains ranged from 0.2 to 1.1 percent, yet differing 1.8 to 2.3 percent from the Chinese strain from the Bohai Sea, over 2.5 to 3.3 percent from *A. peruvianum* morphotype from England, Ireland and Spain. The largest genetic distances were between Baltic Sea and global Atlantic and the Tasman Sea in the South Western Pacific *A. ostenfeldii* strains with a range from 4.4 to 6.1 percent).

### Genetic Structure and Differentiation

**Figure 4:**
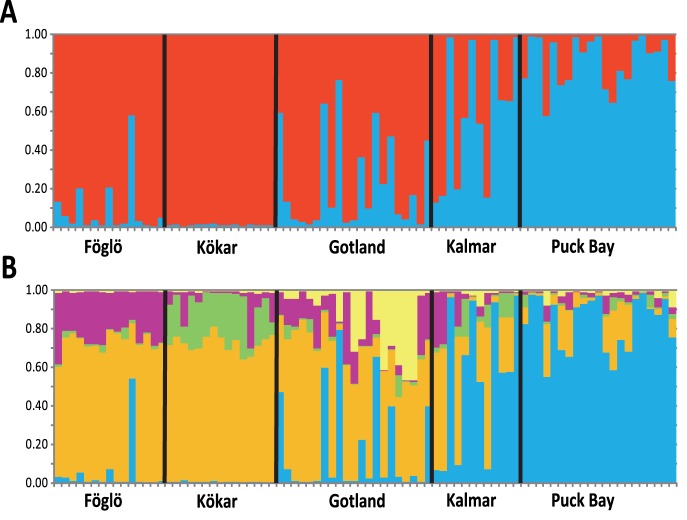
Results from STRUCTURE analyses shown as bar plot of the genotype assignments for 84 *A. ostenfeldii* individuals from five Baltic Sea population samples with (A) *K* = 2, determined as most likely by the method of Evanno et al. (2005), and (B) *K* = 5, determined most likely after Pritchard et al. (2010). Each bar shows the proportional assignment of an isolate’s genome to one of the hypothetical subpopulations. The proportion of each genotype that was assigned to either one of the two hypothetical population subgroups in (A) is “blue” for what was identified as “southern group” and “red” for the “northern group”. In (B) the “southern group” remains unchanged (“blue”), whereas the “northern group” is subdivided into the four additional partitions in the analysis with K = 5 (“purple”, “green”, “orange” and “yellow”). Black vertical lines separate the groups of isolates from the five sampling locations.

Analysis of STRUCTURE results by the Δ*K*-method of [50] gave highest support for two as the most likely number of populations for Baltic Sea isolates of *A. ostenfeldii* (Fig. S1). These two populations grouped the majority of strains according to their geographical origin into a “northern group” and a “southern group”, with almost all strains from Föglö, Kökar and Gotland grouping as one population, and all the strains from Poland and the majority of strains from Kalmar forming the second group (Fig. 4A: the “southern group” is characterized by high proportions of the “blue” genetic component and the “northern group” by prevalence of the “red” genetic partition). Gotland, geographically located in the center of the Baltic proper, and Kalmar in the South were identified as part of a putative hybrid zone, with a relatively high proportion of individuals that were more or less equally assigned to both population subgroups in the STRUCTURE analysis with K = 2 (Fig. 4A).

Following the suggested strategy for inferring the correct number of populations by Pritchard et al. (2010) [52], STRUCTURE results for all values of K were compared and considerable increase in asymmetric assignment of proportions of multilocus genotypes was identified until K = 5 (Fig. 4B). Whereas in simulations with K>2 those proportions of multilocus genotypes assigned to the “southern group” in K = 2 remained unchanged, the “northern group” was subdivided into different partitions. These additional genotype partitions were found in varying proportions, which correspond to the ‘asymmetric’ assignment *sensu* Pritchard et al. (2010) [52]. Most interestingly, these partitions all grouped roughly by geographical origin (e.g. the “green” component was mainly recovered in the population sample from Kökar) – being indicative for capturing real population structure.

**Figure 5:**
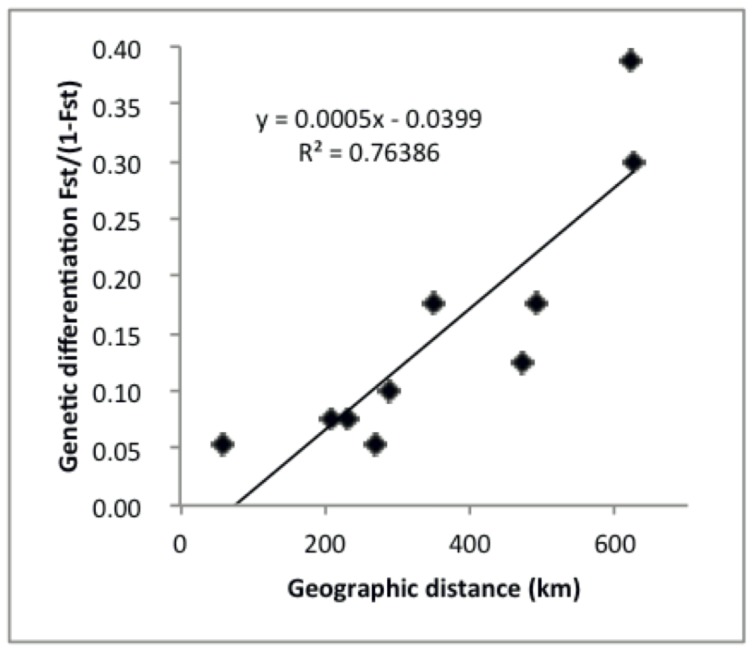
Correlation-analysis of genetic differentiation and geographic distance (Mantel-test based on AFLP-markers) between the studied localities showed high correlation (r^2^ = 0.76) between growing genetic and geographical distance. Results were obtained from program Arlequin 3.5.

The genetic differentiation among the five geographical subpopulations observed in the analysis of STRUCTURE results according to [52] was confirmed by highly significant (p<0.001) *F*_ST_ values for all but one pair-wise comparison after multiple test corrections (Table 1). However, the pair-wise comparison of Gotland and Kalmar still resulted in significant differentiation at p<0.05 (after correction for multiple pairwise tests). The χ^2^- statistic of POWSIM showed that the power of the test on pair-wise differentiation between Kalmar and Gotland was >0.95 at *F*_ST_ = 0.07, indicating a very low likelihood for false negative test results. The largest genetic difference existed between Poland and Åland locations (*F*_ST_ = 0.28 between Kökar and Puck Bay, Poland), whereas, the differences between Föglö and Kökar (*F*_ST_ = 0.05) and Föglö and Gotland (*F*_ST_ = 0.05) were the smallest (Table 1). The relationship between genetic distance and geographical distance of sample sites, as revealed by the Mantel test (Fig. 5), was significantly positive (r^2^ = 0.76, p<0.004) with geographic distance.

**Table 1:** Pairwise genetic distances (*F*_ST_) of five populations of *A. ostenfeldii* from the Baltic Sea estimated from AFLP data (p-values of pair-wise *F*_ST_ were corrected by Bonferroni and Šidák procedures).

Origin	Föglö	Kökar	Gotland	Kalmar
Föglö				
Kökar	0.05**			
Gotland	0.05**	0.09**		
Kalmar	0.11**	0.15**	0.07*	
Puck Bay	0.22**	0.28**	0.15**	0.07**

* p<0.05;

** p<0.001.

### Genetic Diversity within Localities

For the analysis 506 polymorphic loci from a total of 545 were retained. Each band was considered to represent a single locus. The percentage of polymorphic loci ranged from 33% to 59% between localities (Table 2), clearly showing that the most diverse were the northernmost isolates. Also the number of polymorphic loci per locality was highest for isolates from the Åland Sea (Föglö and Kökar). No identical multilocus genotype was found among the 84 isolates of *A. ostenfeldii*, indicating that genotypic diversity (defined as the proportion of unique genotypes) was high, reaching the value of 1 within each of the five population samples as well as the combined Baltic Sea sample. Values of Nei’s gene diversity, indicative for the distribution of allele frequencies averaged over all AFLP loci, were generally low, ranging from 0.07 to 0.11 at the different Baltic locations (Table 2).

**Table 2:** Information on AFLP loci and related metrics (combined from all five primer combinations used) per population and for all Baltic Sea isolates of *A. ostenfeldii* analysed as: PPL (%) = number of polymorphic alleles as percentage of loci, *H* = Neìs gene diversity (Nei 1987), Band presence = average band presence as percentage of genotyped individuals as obtained from AFLPdat (Ehrich 2006), *I* ^s^_A_^ALL^ = standardised index of association as obtained from LIAN 3.5 (Haubold & Hudson 2000).

Origin	PPL (%)	*Ĥ*	Band presences	*I* ^s^_A_^ALL^
Föglö	52	0.10	33.9%	0.0003
Kökar	40	0.09	33.7%	0.0008
Gotland	59	0.11	36.3%	0.0033*
Kalmar	35	0.10	37.7%	0.0039*
Puck Bay	33	0.07	36.9%	0.0037*
Baltic Sea	44	0.10	35.7%	0.0029*

* Significant below p-value 0.01.

### Multilocus Linkage Disequilibrium

Significant multilocus linkage disequilibrium (LD) was detected within population samples from Gotland (AOVA), Kalmar (AOKAL) and Puck Bay, Poland (AOPL) with *I* ^s^_A_ values of 0.0033, 0.0039 and 0.0037 (all p<0.01; Table 2). Multilocus LD for all the joint analysis of all five-population samples taken together was also significant (*I* ^s^_A_ = 0.0029; p<0.01; Table 2).

## Discussion

### Divergence and Phylogenetic Position of Baltic Sea *A. ostenfeldii*

Phylogenetic analyses of the internal transcribed spacer (ITS) sequence data of the rDNA operon revealed a pronounced structure of the *A. ostenfeldii/peruvianum* species complex, resembling the complex phylogenetic relationships among global isolates of other *Alexandrium* species [60]. Generally, these do not support the existing morphological species concepts, but rather suggest the existence of cryptic species with distinct distribution ranges and/or ecological niches. For *A. ostenfeldii/peruvianum* the present phylogenetic analyses resolved six distinct clades. One of these was exclusively comprised by the distinct ITS sequences found in the five investigated Baltic bloom populations. High statistical support for this clade suggests that Baltic Sea *A. ostenfeldii* form a divergent phylogenetic lineage that is clearly separated from all other known global populations of the *A. ostenfeldii/peruvianum* species complex.

In the present ITS phylogeny all sequences derived from Baltic Sea populations group together with that of the isolate from the Bohai Sea (China). Though morphologically largely in accordance with the *A. ostenfeldii* description [30], [61] these strains are genetically clearly differentiated from all other *A. ostenfeldii* isolates including the ones from the Icelandic type location, and from strains morphologically assigned to *A. peruvianum*. The apparent mismatch between morpho- and ribotypes emphasizes the present lack of understanding of the evolutionary relationships in the *A. ostenfeldii*/*peruvianum* species complex and it indicates the incompatibility of molecular phylogenetic evidence with the hitherto applied morphological species concept. The differentiation of several ribotypes within a single morphotype and the intermingling of morphotypes in phylogenetic clades suggest that cryptic species may also exist in the *A. ostenfeldii/peruvianum* complex (for review see [60]). Possibly the cluster containing the Baltic and Chinese strains represents a cryptic, yet undescribed species, which shares physiological characters and ecological preferences and is geographically diverse. For example, unlike most other *A. ostenfeldii and A. peruvianum,* Baltic and Chinese strains do not produce detectable amounts of spirolides, toxins distinctive for *A. ostenfeldii*, but produce saxitoxins or no toxins at all [27], [30], [61]. In contrast to other *A. ostenfeldii/peruvianum* they also share a preference for warm water and the ability to thrive at low salinities. The somewhat counterintuitive clustering of the Chinese together with Baltic isolates may reflect an unrepresentative strain sampling. The *A. ostenfeldii* morphotype has been reported from a number of locations we were not able to cover in the analysis shown here. A more meaningful geographic structure within this ribotype/cryptic species might be revealed when more strains from other locations are included in an analysis aiming at unraveling biogeographic patterns in *A. ostenfeldii* at broad geographic scale. Most importantly, here we conclude on the strict isolation of Baltic Sea *A. ostenfeldii* from geographically adjacent North Sea populations.

The divergence among ITS lineages within the Baltic strains of *A. ostenfeldii* seems to be paradoxical with regard to the timing of colonization of the Baltic and current understanding of the speed of ribosomal sequence evolution in dinoflagellates. The observed differentiation at rDNA level suggests long-standing divergence of the Baltic populations. Even though the rDNA marker used in this study (ITS) is considered to be evolving rapidly compared to 18S rDNA and 28S rDNA, sequence divergence still represents geological rather than ecological time spans according to general expectations. Even neutral mutations resulting from different, local selective regimes are not considered to be expressed rapidly in the rDNA [7]. Although divergence times have not been established for the ITS region, hundreds of thousands to more than millions of years have been discussed as time spans for divergence of ITS sequences in other species of dinoflagellates [62], [63]. According to such assumptions, the level of rDNA differentiation found between the Baltic *A. ostenfeldii* and their geographically neighboring populations from the North Atlantic suggests separation times that by far exceed the postglacial formation of the Baltic Sea ecosystem. Assuming that this degree of genetic divergence represents evolutionary processes on longer time scales, the relatively young Baltic Sea system might have been colonized by dispersing individuals of low salinity adapted populations from other brackish water habitats. Such process would well agree with the idea of large scale dispersal and environmental filtering of microbial populations according to Beijerinck’s hypothesis and its contemporary variants.

However, while the evolution rate of rDNA generally seems to be relatively slow in microbial metapopulations [64], young and small populations like the marginal Baltic Sea *A. ostenfeldii* might have accelerated divergence rates, which could have led to the observed pattern. These, in turn are most likely explained by strong effects of neutral and/or adaptive processes governed by the very specific environmental regime in the Baltic Sea. Such conditions may even support the evolution of new species as shown by the case of the brown algae *Fucus radicans,* which has diverged from *Fucus vesiculosus* well after the postglacial opening of the Baltic Sea within only a few hundred years [65].

It has, furthermore, been suggested that the rise of genetically unique and highly differentiated Baltic populations of widely distributed bivalve species may be a result of postglacial hybridization of long time isolated, geographically distant lineages that were naturally dispersed into the newly opened Baltic Sea. In such lineages processes of genetic differentiation seem to be significantly faster than it would generally be expected from neutral mutation rates [66]. Interestingly, the demonstrated ITS lineage divergence among strains of *A. ostenfeldii* from the Baltic Sea is in contrast to the pattern shown for the congeneric dinoflagellate *A. minutum* in the Mediterranean Sea, where ITS ribotypes in a large number of isolates were identical despite the presence of clear population differentiation as assessed by polymorphic microsatellite loci [67].

Although the present data support the concept of a distinct Baltic lineage that diverged due to adaptation to low salinity conditions of the Baltic Sea after the last glaciations, it cannot be excluded that Baltic *A. ostenfeldii* may have been selected through environmental filtering from a global pool of brackish-adapted populations not covered by the present strain sampling. More extensive representation of global strains including samples from other brackish-water habitats may reveal that monophyly of the Baltic clade is not provided which would imply that the adaptation to brackish water could have arisen long before the appearance of the Baltic Sea, and the Baltic lineage would have derived from dispersal.

### Genetic Structure in the Baltic Sea and Implications for Gene Flow and Expansion

The distribution of divergent ITS lineages within the Baltic *A. ostenfeldii* clade does not reflect an obvious differentiation pattern. ITS haplotype network analyses revealed that the two major haplotypes were present in northern, central as well as southern bloom sites. The only exception is Kalmar, which includes a unique haplotype represented by one third of the strains analyzed from that population, but lacks another haplotype that is shared by all other populations sampled in this study. This could be interpreted as a indication of weak genetic differentiation. Apparently the level of gene flow among the majority of Baltic bloom populations is too high to generate a clear signature of differentiation at the rDNA level.

As expected, a more obvious differentiation pattern was revealed by the analysis of >500 independent AFLP markers. Here the two hypothetical genetic population subdivisions identified by STRUCTURE according to the ΔK-method are predominantly related to geographic locations in the northern (Föglö and Kökar) and central (Gotland) Baltic or to southern locations (Kalmar and Puck Bay, Poland) (see Fig. 4 A) and B) where genotype proportion of the “southern group” are shown in “blue” fractions of the bar plots). The Gotland population in the central Baltic proper was identified as being part of a potential hybrid zone, characterized by a relative high proportion of genotypes with mixed ancestry. The high proportion of individuals sharing hypothetical origin with the majority of individuals from the northern population samples from Föglö and Kökar, however, indicate a closer genetic affiliation with these populations. AFLP data revealed highly significant genetic differentiation, as measured by pairwise *F*_ST_, among all studied Baltic subpopulations, except for the pairwise analysis between Gotland and Kalmar where statistical support was considerably lower though still significant at p<0.05. Interestingly, the two methods for inference of the number of genetic population subgroups applied in this study yielded different results. These findings may indicate that the Δ*K*-method tends to identify the largest genetic break among hypothetical population subgroups, whereas the method proposed by Pritchard et al. 2010 [52] allows to detect more subtle population genetic structure below the uppermost hierarchical level. Indeed it has been reported that evaluation of STRUCTURE analyses based on the Δ*K*-method in some cases, especially when relatively weak genetic differentiation among geographically separated populations must be assumed, tends to not reveal the finest underlying population structure [68].

Hence, the origin of the genetic differentiation among those populations with similar proportions of individuals assigned to either the “northern group” or the “southern group” in *K* = 2, becomes more evident in the STRUCTURE assignment pattern with *K* = 5. Whereas the “southern group” does not show further subdivision, the “northern group” divides into additional subgroups. By displaying a geographical prevalence these subgroups allow for identifying a pattern of more subtle genetic differentiation and gene flow. Whereas the dominant proportion of the subdivided “northern” genetic component (“orange” in Fig. 4B) is present in the majority of multilocus genotypes, the smaller components (“purple”, “green”, “yellow” in Fig. 4b) show population specific partitioning of genetic components. These genetic partitions explain the highly significant genetic differentiation by *F*_ST_ found e.g., for the closely located northern population samples from Föglö and Kökar, which cannot be explained by the grouping according to the STRUCTRUE analysis with *K* = 2 (see Fig. 4 A) and B). In the population sample from Gotland the population specific genetic component evident in the STRUCTURE analysis with *K* = 5 (“yellow” in Fig. 4B), however, only is displayed by about half of the genotypes. A high proportion of genotypes similar to those from Föglö suggest that relatively high gene flow also occurs from Föglö to Gotland, but less so in the other direction (as no genotypes from Föglö display the signature of the specific genotypic component (“yellow” in Fig. 4B) found in some of the AOVA strains from Gotland). The observed genetic differentiation pattern might reflect an established but balanced differentiation with low to moderate levels of asymmetric gene flow among spatially restricted locations. The strong correlation between the observed genetic and geographical distances, as well as increasing levels of genetic differentiation and low genotypic variation between the subgroups support this idea.

The genetic substructure suggested by multilocus genotype assignment data for Baltic *A. ostenfeldii* seems to be linked to the current regime prevailing in the Baltic Sea and possibly also to life cycle peculiarities of the organism as reasoned in the following. Gene flow between the southern, Puck Bay, Poland and Kalmar) and northern populations can be expected to be limited, since these bloom locations are not immediately connected by the major circulation pattern: From the Polish coast, the major currents move along the southern coasts northeastwards up into the Gulf of Finland. The currents passing Åland, on the other hand, predominantly take northern or western directions before they join the coastal currents moving southwards between Gotland and the coast of Sweden [32]. Such a pattern is likely to maintain oceanographic advection of planktonic populations from bloom locations in Kökar, Föglö and Gotland and support admixture among these bloom sites as well as the directional gene flow as indicated by the STRUCTURE analysis. Low levels of genetic differentiation as evident from *F*_ST_ values obtained from AFLP among Kökar, Föglö and Gotland might therefore be explained by relatively high rates of gene flow. The Coastal Swedish current, in turn, is likely to facilitate gene flow from Gotland to Kalmar. The presence of “southern” genotypes in the Gotland bloom location despite the North-South direction of the major current flow may be explained by transport through an internal circulation system of the Central Baltic Proper, which surpasses the loop of eastwards coastal currents into to Gulf of Finland and re-enters the western current after passing the northern tip of Gotland. This internal loop directly connects the southern locations (Kalmar and Puck Bay, Poland) with central Gotland.

A more or less continuous gradient of genetic differentiation seems to exist among the local populations, which even is evident in the closely located, but still weakly differentiated population samples from Kökar and Föglö. Whereas the overall genetic structure according to Δ*K* of [50] does not allow distinguishing between these two populations, subtle but highly significant genetic differentiation (*F*_ST_ = 0.05, p<0.001 after correction) is evident in the STRUCTURE results from *K* = 5 with genetic partitions uniquely found in both populations (see Fig. 4B). Hence, it is possible that Baltic *A. ostenfeldii* is structured as a result of early post-glacial colonization of the Baltic Sea, where established populations have gone through fast local adaptation and remained mostly isolated due to limited dispersal between subpopulations. In fact, the studied populations are all from similar coastal sheltered environments where blooms are localized phenomena, i.e. they are not typically dispersed far beyond the restricted bloom areas [3]. This means that the subpopulations most likely remain anchored in their bloom locations by their cyst beds. As shown for the coastal diatom *Skeletonema marinoi,* tight coupling between propagule banks and planktonic blooms can generate and maintain locally adapted endogenous populations [6]. Typically, dinoflagellate resting cysts are formed by sexual processes e.g. [31]. Such should be particularly effective in blooms where the density necessary for the successful encounter of gametes is provided. Outside the local embayments such cell densities might never be reached and thus it is unlikely that seedbeds are continuous between study sites. Such circumstances would counteract dispersal and gene flow among the subpopulations as expected from large-scale Baltic Sea surface water circulation patterns.

Our findings on *A. ostenfeldii* differentiation in the Baltic Sea are generally in accordance with the patterns observed in higher organisms. Coastal Baltic macrophytes [23], invertebrates [17] and fish [71] are generally characterized by high population differentiation, even over very short distances. Isolation by distance was shown for the brown algae *Fucus vesiculosus* and, similar to what is discussed for *A. ostenfeldii*, related to reproduction strategies, i.e. release of sexual cells that typically serve as dispersal propagules during calm water preventing dispersal and gene flow [29]. Physical barriers have been suggested as a reason for differences in the genetic structure of *Perca fluviatilis* in different Baltic subbasins [69]. The specific life cycle peculiarities of meroplanktonic dinoflagellates such as *A. ostenfeldii*, however, certainly add specific constraints to the genetic differentiation in this species and other patterns of genetic differentiation might be found eukaryotic microalgae that do not (or to lesser extent) depend on suitable sites for cyst deposition.

### Genetic Diversity of Baltic Sea *A. ostenfeldii* at Basin and Local Scale

Although direct comparison between Baltic and North Atlantic populations of *A. ostenfeldii* is not possible due to the lack of sufficient isolates for AFLP analyses from the latter, comparison with adequate data for other dinoflagellate species [10], [63] suggests that gene diversities measured in Baltic *A. ostenfeldii* (ranging from 0.07 to 0.11) are generally lower. For example, the gene diversity reported for an Atlantic *A. tamarense* population – a species that does not exist in the Baltic Sea – was twice as high as that of Baltic Sea *A. ostenfeldii* [10]. Interestingly, low levels of genetic diversity – comparable to Baltic Sea *A. ostenfeldii* – have been detected in *Polarella glacialis* and *Scrippsiella* aff. *hangoei* populations from saline Antarctic lakes [63], which also represent ecologically extreme habitats. Low genetic diversity has been shown for a number of macroscopic Baltic Sea organisms such as seagrasses [70], bivalves [22] or fishes [69], [71] – and has been interpreted as a consequence of life under extreme environmental conditions, particularly the very low salinities [14]. It is possible that also the low diversity levels found in the Baltic Sea *A. ostenfeldii* populations are attributed to the ecologically extreme and geographically marginal habitat conditions, the species experiences in the central and northern Baltic Sea.

Besides population genetic factors such as bottlenecks and genetic drift, other factors that have been proposed to cause a decrease in gene diversity include the loss of sexual reproduction or shifts towards a higher contribution of asexual reproduction. In organisms such as *A. ostenfeldii*, in which life cycle strategies combine sexual and asexual reproduction modes, such phenomena might be especially common. Association analyses, an approach that is commonly used to assess reproduction modes in microbial organisms, where sexuality cannot be observed directly [72], revealed significant multilocus linkage disequilibrium (LD) – expressed as *I* ^s^_A_ values significantly different from zero – in the southern and central subpopulations (Poland, Kalmar, Gotland), which indicates a strong influence of asexual reproduction.

This finding could be the result of an atypical life cycle strategy with prevalence of asexual cysts instead of the typical sexual resting stages *A. ostenfeldii/peruvianum* can produce asexual resting cysts [31] and may behave similarly to the Baltic cold water than dinoflagellate *Scrippsiella hangoei,* which mainly encysts without prior sexual zygote formation [73]. In the Baltic Sea, loss of sexuality has been repeatedly demonstrated for higher organisms such as seagrasses and brown algae and been suggested to result from physiological stress experienced by marine species at the prevailing low salinities. This mechanism may also act in Baltic microorganisms, but more conclusive data is necessary to substantiate it.

Low incidence of recombination as suggested by significant LD may also be a result of homothallism and high selfing rates [74] or different net growth capabilities of the individual genotypes causing relative dominance of only a few clonal lineages in the plankton and thereby leading to a genetically impoverished and differentiated cyst pool derived from such planktonic populations. Differences in the effect of selective regimes in southern, central and northern populations may sustain the differences in *I* ^s^_A_ as their specific signature of selection. The last of these possible causes of multilocus LD has been proposed to explain findings in the congeneric species *A. tamarense* in a detailed study on a planktonic population of this species from the North Sea [11].

In addition to the above detailed causes for intrinsic generation of multilocus LD within a local population, admixture LD caused by admixture of individuals from other genetically differentiated populations might explain within population multilocus LD especially in the case of the population sample from Gotland, where the most complex pattern of within population genetic structure was observed. Here, similar to the basin wide detected multilocus LD, the observed deviation from an independent distribution of alleles within multilocus genotypes might predominantly result from the simultaneous analysis of multilocus genotypes originating from genetically differentiated populations. The (relative) contribution of one of the possible causes for multilocus LD, however, cannot be specified with the data at hand and more intensive studies of processes at the within population level are certainly needed to unravel the significance of intrinsic population genetic processes in meroplanktonic dinoflagellates such as *A. ostenfeldii*.

Despite low general gene diversity levels, which might be caused by population genetic or environmentally driven processes, genotypic diversity within each of the Baltic subpopulations was high. None of the local subsets of isolates contained identical genotypes. Moreover, not a single genotype was repeatedly sampled in the total set of 84 Baltic Sea *A. ostenfeldii* analyzed in this study. This confirms what has so far been shown by most other studies on marine microalgae using markers with enough differentiating capacity (see e.g. [10]. Such variability of genotypes – if representative for adaptively significant genetic variation – may explain the phenotypic differences with regard to morphological characters, salinity tolerances and their response to the climate stressors [30], [75].

### Conclusions

This study presents, for the first time, data on the genetic structure of a eukaryotic microorganism from the Baltic Sea. Our results show that the Baltic population of the toxic dinoflagellate *Alexandrium ostenfeldii* is phylogenetically distinct from all other known global lineages of the species and micro-geographically fragmented within the Baltic Sea. Significant genetic differentiation between northern and southern locations exists, with increasing genetic differentiation with geographical distance. Local subpopulations generally exhibited low levels of overall gene diversity despite the high genotypic diversity that was found among individual strains. Association analyses suggest that predominantly asexual reproduction and resulting clonality may contribute to high levels of multilocus linkage disequilibrium. Our results indicate that the general pattern of genetic differentiation and reduced genetic diversity of Baltic populations found in large organisms to some extent also applies to microscopic eukaryotic organisms, which emphasizes that the mechanisms driving these patterns are effective in Baltic microorganisms, despite their very different organism properties, life history traits and habitat conditions. Moreover, the unexpected pattern of ribotypic diversity and differentiation among the studied populations may indicate that evolutionary processes are manifesting their signatures much faster in the Baltic Sea than in other – non-marginal – environments.

## Supporting Information

Figure S1 **Most probable number of genetic populations as determined from STRUCTURE (2.3.3.) analysis 84 individual** ***A. ostenfeldii*** **AFLP-genotypes.**
(TIF)

Table S1 **Sampling area and rate of culture survival.**
(DOCX)

Table S2 **Information on strains and species used for ITS phylogenetic analysis.**
(DOCX)

Table S3 **Description of AFLP primer characteristics.**
(DOCX)

Table S4 **Genetic difference and distance between populations based on ITS regions (572 bp).**
(DOCX)

Text S1 **AFLP reaction protocol.**
(DOCX)
