# Supplementary figures and images for: Patterns of Post-Glacial Genetic Differentiation in Marginal Populations of a Marine Microalga
Source: PLoS One. 2012 Dec 31;7(12):e53602. doi: 10.1371/journal.pone.0053602 (PMC3534129; doi:10.1371/journal.pone.0053602)

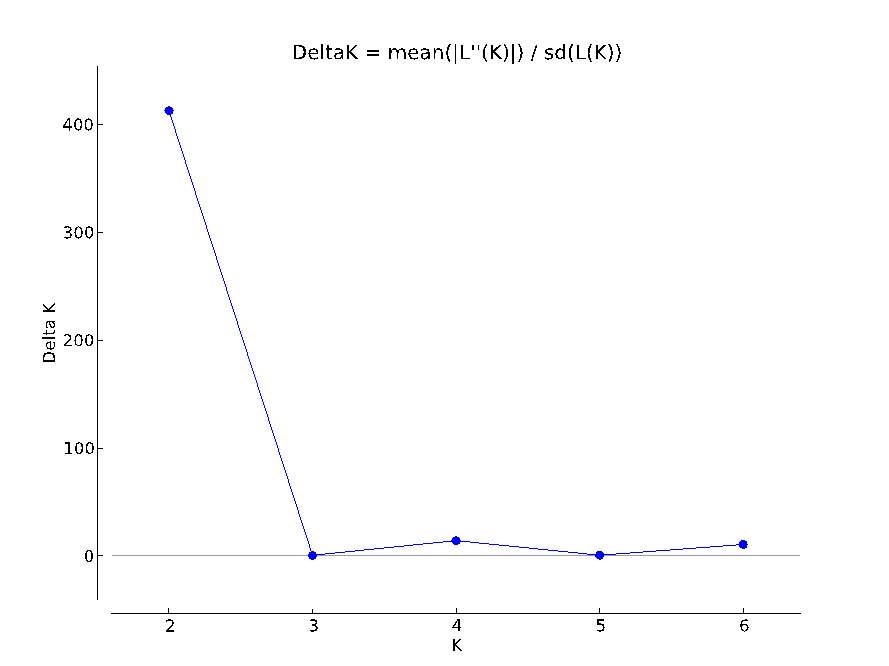

Supplement: Figure S1 — Most probable number of genetic populations as determined from STRUCTURE (2.3.3.) analysis 84 individual A. ostenfeldii AFLP-genotypes. (TIF) [file pone.0053602.s001.tif]
