# Supplementary material for: Patterns of Post-Glacial Genetic Differentiation in Marginal Populations of a Marine Microalga
Source: PLoS One. 2012 Dec 31;7(12):e53602. doi: 10.1371/journal.pone.0053602 (PMC3534129; doi:10.1371/journal.pone.0053602)
Supplement: Table S1 — Sampling area and rate of culture survival. (DOCX) [file pone.0053602.s002.docx]

| Table S1. Sampling area and rate of culture survival | | | | |
| --- | --- | --- | --- | --- |
| Sampling area | Year | Strains | Survival rate % |  |
| Föglö, Finland | 2009 | 15 | 59.4 |  |
| Kökär, Finland | 2010 | 15 | 71.0 |  |
| Gotland, Sweden | 2009 | 21 | 41.5 |  |
| Kalmar, Sweden | 2009 | 12 | 34.9 |  |
| Puck Bay, Poland | 2009 | 21 | 39.4 |  |
| Survival rate of total |  | 84 | 51.6 |  |
