# Supplementary material for: Patterns of Post-Glacial Genetic Differentiation in Marginal Populations of a Marine Microalga
Source: PLoS One. 2012 Dec 31;7(12):e53602. doi: 10.1371/journal.pone.0053602 (PMC3534129; doi:10.1371/journal.pone.0053602)
Supplement: Table S2 — Information on strains and species used for ITS phylogenetic analysis. (DOCX) [file pone.0053602.s003.docx]

| Species | Strain | Origin | Reference | Acc. No |
| --- | --- | --- | --- | --- |
| *Alexandrium ostenfeldii* | AOF0905 | Åland, Finland | this study | JX865526 |
| *Alexandrium ostenfeldii* | AOF0917 | Åland, Finland | this study | JX865531 |
| *Alexandrium ostenfeldii* | AOK1005 | Åland, Finland | this study | JX865527 |
| *Alexandrium ostenfeldii* | AOK1017 | Åland, Finland | this study | JX865532 |
| *Alexandrium ostenfeldii* | AOVA0901 | Gotland, Sweden | this study | JX865529 |
| *Alexandrium ostenfeldii* | AOVA0924 | Gotland, Sweden | this study | JX865536 |
| *Alexandrium ostenfeldii* | AOVA0925 | Gotland, Sweden | this study | JX865535 |
| *Alexandrium ostenfeldii* | AOKAL0902 | Kalmar, Sweden | this study | JX865533 |
| *Alexandrium ostenfeldii* | AOKAL0909 | Kalmar, Sweden | this study | JX841280 |
| *Alexandrium ostenfeldii* | AOKAL0913 | Kalmar, Sweden | this study | JX865530 |
| *Alexandrium ostenfeldii* | AOPL0915 | Hel, Poland | this study | JX865534 |
| *Alexandrium ostenfeldii* | AOPL0917 | Hel, Poland | this study | JX841277 |
| *Alexandrium ostenfeldii* | AOPL0962 | Hel, Poland | this study | JX865528 |
| *Alexandrium peruvianum* | IEO-VGOAMD12 | Palamos, Spain | this study | JX841266 |
| *Alexandrium peruvianum* | IEO-VGOAM10C | Palamos, Spain | this study | JX841267 |
| *Alexandrium ostenfeldii* | ASBH01 | Bohai Sea, China | Gu 2012 | JN173268 |
| *Alexandrium peruvianum* | WW516 . | Fal River, UK | this study | JX841256 |
| *Alexandrium peruvianum* | WW517 | Fal River, UK | this study | JX841255 |
| *Alexandrium peruvianum* | LSA06 | North Sea, Ireland | this study | JX841261 |
| *Alexandrium ostenfeldii* | AONOR4 | Oslofjord, Norway | this study | JX841279 |
| *Alexandrium ostenfeldii* | NCH85 | North Sea, Norway | this study | JX841259 |
| *Alexandrium ostenfeldii* | CCMP1773 | Limfjord, Denmark | Orr et al. 2011 | JF521636 |
| *Alexandrium ostenfeldii* | CCAP1119/45 | North Sea, Scotland | this study | JX841272 |
| *Alexandrium ostenfeldii* | AONS = S6_P12_E11 | North Sea, Scotland | this study | JX865537 |
| *Alexandrium ostenfeldii* | AOIS4 | Breidafjord, Iceland | this study | JX841281 |
| *Alexandrium ostenfeldii* | LKE6 | Gulf of Maine, USA | this study | JX841262 |
| *Alexandrium ostenfeldii* | F301 | Gulf of Maine, USA | this study | JX841270 |
| *Alexandrium ostenfeldii* | CAWD135 | New Zealand | this study | AB753843 |
| *Alexandrium ostenfeldii* | CAWD136 | New Zealand | this study | AB753844 |
| *Alexandrium insuetum* | CSIS-1 | Spain | Penna et al. 2008 | AM422769 |
| *Alexandrium tamutum* | IEO-VGO-662 | Italy | Penna et al. 2008 | AM238452 |
| *Alexandrium minutum* | IEO-AL-10C | Spain | Penna et al. 2008 | AJ620856 |
