# Supplementary material for: Patterns of Post-Glacial Genetic Differentiation in Marginal Populations of a Marine Microalga
Source: PLoS One. 2012 Dec 31;7(12):e53602. doi: 10.1371/journal.pone.0053602 (PMC3534129; doi:10.1371/journal.pone.0053602)
Supplement: Table S3 — Description of AFLP primer characteristics. (DOCX) [file pone.0053602.s004.docx]

| Primer combination | *Eco*RI primer | *Mse*I primer | Loci | Error rate |
| --- | --- | --- | --- | --- |
| PC1 | +TCG | +CGC | 100 | 4.9 |
| PC2 | +TG | +CGG | 150 | 3.9 |
| PC4 | +AAG | +CGC | 85 | 3.2 |
| PC5 | +TG | +CGA | 75 | 2.5 |
| PC7 | +TC | +CGC | 135 | 6.9 |
