# Supplementary material for: Patterns of Post-Glacial Genetic Differentiation in Marginal Populations of a Marine Microalga
Source: PLoS One. 2012 Dec 31;7(12):e53602. doi: 10.1371/journal.pone.0053602 (PMC3534129; doi:10.1371/journal.pone.0053602)
Supplement: Table S4 — Genetic difference and distance between populations based on ITS regions (572 bp). (DOCX) [file pone.0053602.s005.docx]

| Population | Difference | Distance |
| --- | --- | --- |
| Föglö | 0 | 0 |
| Kökar | 0 | 0 |
| Gotland | 0 | 0 |
| Kalmar | 3 | 0.005 |
| Poland, Puck Bay | 2 | 0.003 |
| China | 10 | 0.017 |
| England (WW516/WW517) | 14 | 0.024 |
| Mediterranean (IEO-D12/IEO-10C) | 16 | 0.028 |
| Ireland (IRH6) | 14 | 0.024 |
| Gulf of Maine (F302) | 27 | 0.047 |
| Gulf of Maine (LKE6) | 26 | 0.045 |
| Iceland (AOIS4) | 27 | 0.047 |
| Norway (AONOR4) | 32 | 0.056 |
| Norway (AONS) | 32 | 0.056 |
| Scotland (CCAP1119/45) | 32 | 0.056 |
| Scotland (NCH85) | 32 | 0.056 |
| New Zealand (CAWD135) | 29 | 0.051 |
