# Supplementary material for: Patterns of Post-Glacial Genetic Differentiation in Marginal Populations of a Marine Microalga
Source: PLoS One. 2012 Dec 31;7(12):e53602. doi: 10.1371/journal.pone.0053602 (PMC3534129; doi:10.1371/journal.pone.0053602)
Supplement: Text S1 — AFLP reaction protocol. (DOCX) [file pone.0053602.s006.docx]

AFLP reaction protocol

AFLP fragments were generated using the PCR conditions previously described in Vos et al. (1995) and Figueroa et al (2010), with exceptions that DNA was digested for 17 hours at 37°C with MseI and EcoRI restriction endonucleases and dilutions for pre- and selective-amplification were done in five fold. Digestion mixture per sample contained 3.45 µl of sterile ddH20, 1 µl Buffer4 (10x, NEB, New England Biolabs), 0.5 µl BSA (NEB), 0.125 µl MseI (10 U) (NEB, New England Biolabs), 0.05 µl EcoRI (NEB, New England Biolabs), and 50 ng of diluted DNA. After digestion, restriction endonucleases were heat-inactivated on ice for 15 minutes. Genomic DNA fragments were ligated for 3 hours in 37 C. Success of digestion was checked on agarose gel (2%), and digested DNA was diluted 1:5 before ligation reactions. Ligation was performed in 12.5µl mixture containing 0.0125 µl 100 µM EcoRI-adaptor (all oligonucleotides used were ordered from Oligomer Oy, Finland) and 0.125 µl 100 µM MseI-adaptor, 0.25 µl Ligation buffer (10x, NEB), 2.06 µl sterile ddH20, 0.05 µl T4-ligase (5U, NEB) and 10 µl of diluted and digested DNA. After ligation samples were diluted (1:5). Pre-amplification mixture contained 0.9 µl sterile ddH20, 1 µl MgCl2 (25 mM, FastStart, Roche), 1 µl PCR-buffer (10x, FastStart, Roche), 2 µl dNTP (1mM, Fermentas), 0.03 µl E-primer (100 µM), 0.03 µl M-primer (100 µM), 0.04 µl Taq (5U, FastStart DNA Polymerase, Roche) and 5 µl diluted DNA from ligation. DNA was amplified with pre-amplification primers (E+0, M+0) in PCR-conditions described in Vos et al. 1995. After pre-amplification, PCR-products were diluted (1:5) and used as a template for the selective amplification using six different primer pair combinations (Table 3), each containing two to three selective nucleotides. Selective amplifications were performed with PCR-conditions described in Vos et al. 1995 in 10 µl reaction volumes containing 2.5 µl DNA template and 7.5 µl of reaction mixture comprising 3.3 µl ddH20, 1 µl 25 mM MgCl2 (FastStart, Roche), 1 µl PCR buffer (10×) (FastStart, Roche), 2 µl 1 mM dNTP (Fermentas), 1 µl Taq-DNA polymerase (5 U, FastStart, DNA polymerase, Roche), 0.06 µl 100 µM FAM-labelled EcoRI primer and 0.06 µl 100 µM MseI primer. The final PCR products were separated in capillary electrophoresis using an ABI 3730XL (Applied Biosystems) in Biotechnology Institute (Helsinki, Finland) with Genescan 500kb as internal size standard in each run.
